# Combined effect of early diagnosis and treatment on the case fatality risk of COVID-19 in Japan, 2020

**DOI:** 10.1038/s41598-023-33929-y

**Published:** 2023-04-24

**Authors:** Yuri Amemiya, Hiroshi Nishiura

**Affiliations:** School of Public Health, Kyoto University, Yoshida-Konoe-cho, Sakyo-ku, Kyoto, 606-8501 Japan

**Keywords:** Computational biology and bioinformatics, Public health, Viral infection

## Abstract

Japanese government initially enforced restrictions on outpatient attendances among febrile individuals suspected of having COVID-19, asking everyone to remain at home for at least 4 days from the onset of fever. This restriction was cancelled on 8 May 2020, and a new antiviral, remdesivir, was approved from 7 May 2020. To investigate how this policy change influenced the prognosis of people with COVID-19, we estimated the case fatality risk as a function of the date of illness onset from April to June 2020. We used an interrupted time-series analysis model with an intervention date of 8 May 2020, and estimated time-dependent case fatality risk by age group. The case fatality risk showed a decreasing trend in all groups, and models were favored accounting for an abrupt causal effect, i.e., immediate decline in fatality risk. The trend was estimated at − 1.1% (95% CI [confidence interval]: − 3.9, 3.0) among people aged 60–69 years, − 7.2% (95% CI − 11.2, − 2.4) among those aged 70–79 years, − 7.4% (95% CI − 14.2, 0.2) among those aged 80–89 years, and − 10.3% (95% CI − 21.1, 2.7) among those aged 90 and over. Early diagnosis and treatment greatly contributed to reducing the case fatality risk.

## Introduction

As of 7 March 2023, there have been 6,866,434 documented deaths from COVID-19 worldwide^1^. The disease involves severe manifestations among older people and those with underlying comorbidities, and the life expectancy at birth in the United States has been shortened by 2 years by the pandemic^2,3^. Chronic diseases including diabetes mellitus, chronic obstructive lung disease and acute kidney dysfunction, and other factors including older age, smoking and obesity, are known to elevate the risk of death from COVID-19^4–6^. The World Health Organization (WHO) has stated that the end of the COVID-19 pandemic is in sight in late 2022^7^, but the disease is likely to continue to kill a vulnerable fraction of the population and remain a leading infectious disease problem across the world for some time.

Individual factors may affect the risk of death from COVID-19, but this risk is also known to vary with public health and social measures such as lockdown and shelter-in-place orders^8,9^. These measures reduce the incidence of COVID-19 infection and therefore result in reduced mortality at a population level^10^. However, the case fatality risk is also reduced by decreased incidence, because access to healthcare services has sometimes been hampered by increased case load demand^11^. To minimize the mortality burden of COVID-19, it is vital to understand how the risk of death varies with interventions.

The incidence was very low in Japan during the first wave of COVID-19 from February to May 2020^12^, however the Japanese government imposed restrictions on outpatient visits from 17 February 2020^13^. The clinical condition of working-age pneumonia patients was frequently exacerbated by delayed diagnosis. In Japan, the death of a famous actress was widely attributed to this^14^. However, specific treatments including antivirals were not widely available during the very early stages of the first wave.

Early diagnosis affects the prognosis of COVID-19^15^. Regardless of the presence of specific treatments for viral pneumonia, early treatment of acute respiratory distress syndrome leads to an improved course of illness^16,17^. When the restriction on outpatient visits was imposed in Japan during the first wave, Japan’s Ministry of Health, Labour and Welfare (MHLW) defined indications that should lead people to seek treatment for suspected COVID-19^13^. In the northern hemisphere, the COVID-19 pandemic started in the middle of winter, overlapping with seasonal influenza, and the Japanese government therefore intended to cover treatment only among those who were highly likely to have COVID-19. However, the restriction was cancelled on 8 May 2020, after concerns were raised about the exacerbation of symptoms by delayed diagnosis, and when the influenza season was recognized as over^18,19^. Around the same time, remdesivir (an antiviral drug that is an RNA polymerase inhibitor, recognized as a nucleotide prodrug of an adenosine analog) was officially approved by the government for the treatment of pneumonia caused by SARS-CoV-2^20^, while clinical efficacy of remdesivir was debated in early 2020^21,22^. Due to limited supply of remdesivir before the approval, it was mandatory for each healthcare facility to apply for an approval by MHLW to prescribe remdesivir^23^, and it was unlikely that remdesivir is commonly prescribed to patients with COVID-19. Following the approval this specific treatment became widespread for pneumonia patients, and the government rapidly began to promote early diagnosis and specific treatment of COVID-19, potentially contributing to reducing the case fatality risk.

A sudden change promoting early diagnosis and treatment could have significantly varied the risk of death from COVID-19 in Japan, offering an important research opportunity to estimate the causal impact of the policy on case fatality risk. This study aimed to estimate the causal effect of the state of emergency and early diagnosis and treatment in reducing the case fatality risk during the first wave of COVID-19 from April to June 2020, using interrupted time-series analysis.

## Methods

### Epidemiological data of cases and deaths

COVID-19 has been a designated infectious disease in Japan under Infectious Disease Law since 1 February 2020, and physicians must notify confirmed cases within 24 h of diagnosis^24^. Confirmatory diagnosis is made by the detection of SARS-CoV-2 using reverse transcriptase PCR (RT-PCR) testing^25^. This type of testing was exclusively used during the first wave. In this study, we analyzed notification data of confirmed and deceased patients that were openly announced by 47 prefectures and collected by the MHLW. We collected information about age, the date of illness onset, the date of reporting, and outcome for each patient (i.e., recovered or died). Age data were grouped into 10-year cohorts up to and including those aged 80–89 years, and all those aged 90 years and older were grouped together.

**Figure 1:**
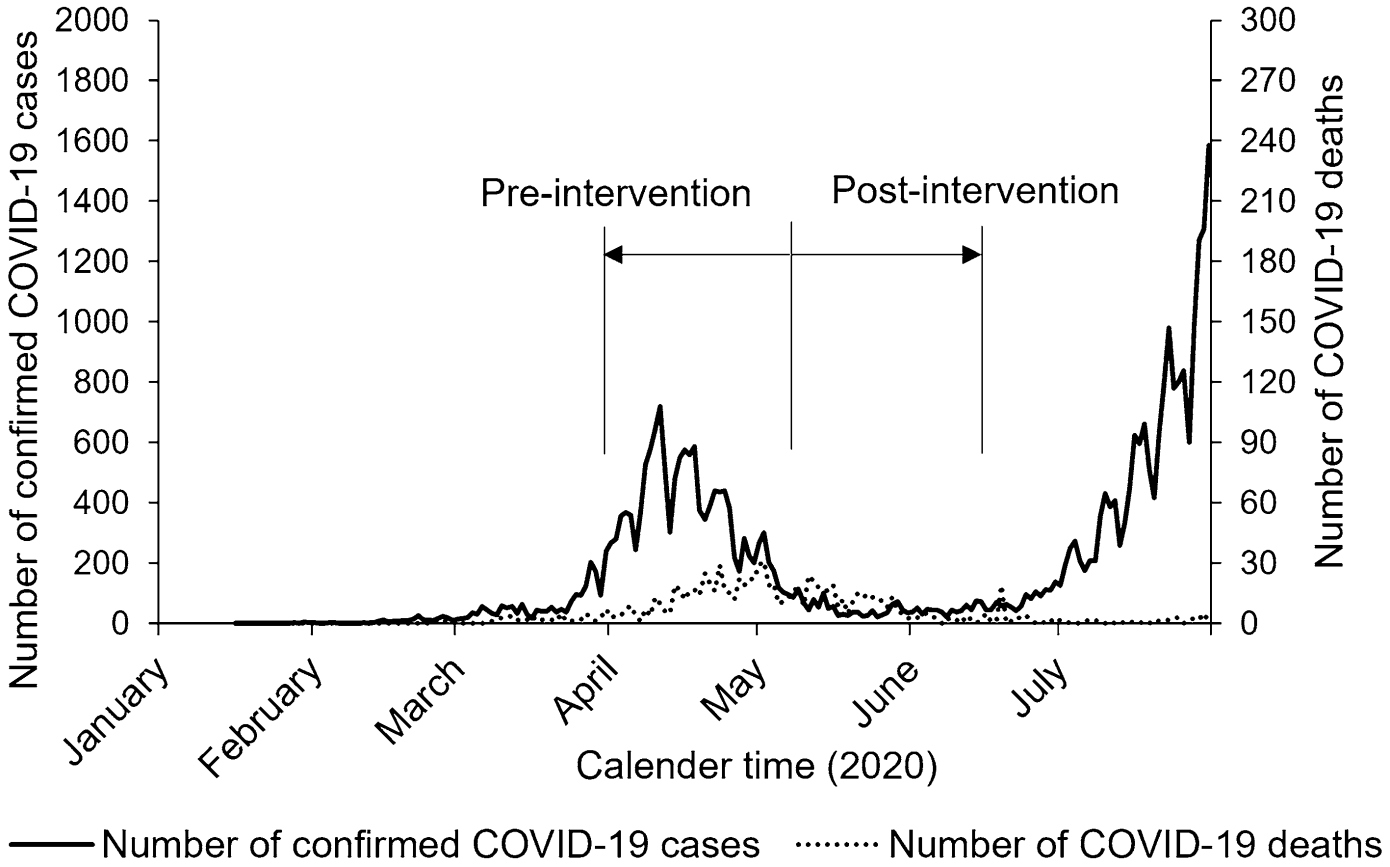
Temporal distributions of confirmed COVID-19 cases and deaths in Japan, January to July 2020. The horizontal axis shows the calendar date of reporting (all cases) and death (deceased individuals). All confirmed cases undertook RT-PCR testing during the corresponding time period. The left vertical axis represents the number of confirmed COVID-19 cases, and the right vertical axis the number of COVID-19 deaths. Within the panel, there are three solid vertical lines, showing the time period of our analysis. The left vertical line is the start date of the study period (1 April 2020), the center is the start date of improved diagnosis and treatment (8 May 2020), and the right is the end date of the study period.

Figure 1 shows the epidemic curve of cases and deaths during the first wave in Japan. A surge of cases was seen from late March 2020, and a state of emergency was declared on 7 April 2020 for seven prefectures out of the total of 47, and people were asked to reduce contact by 80% and stay at home^26^. Due to continued spread, the state of emergency was declared for all remaining prefectures in Japan on 16 April 2020. Incidence as a function of reporting date started to decrease by late April, and the state of emergency was cancelled at the end of May.

### Statistical model to estimate the case fatality risk

We compared two pieces of information, the time from illness onset to diagnosis and the case fatality risk, before and after 7 April and 8 May 2020, respectively. For the former metric, we fitted a gamma distribution to the number of days from illness onset to diagnosis^27^. As well as comparing it before and after 8 May, we also estimated the distribution among survivors and those who died.

The case fatality risk was also estimated as a function of the date of illness onset. If the date of illness onset was missing (38.1% of cases), we deterministically back-projected the date of illness onset by subtracting the mean number of days from onset to diagnosis from the date of diagnosis. Let 7 April 2020 be the “first intervention” date and 7 May 2020 be the “second intervention” of the interrupted time-series analysis. The pre-intervention period was set as 14 March to 6 April 2020, because the surge of cases started to be seen from late March. The first intervention period was 7 April to 7 May 2020, and the second intervention period was 8 May to 15 June, approximately the same length as the pre-intervention and first intervention periods. Case fatality risk was calculated as the number of deaths as a fraction of the number of confirmed cases^28^. This frequently took extremely small values among working-age adults, and the score confidence interval was therefore computed as:

1 $$\left( {\hat{p} + \frac{{z_{{{\raise0.7ex\hbox{$\alpha $} \!\mathord{\left/ {\vphantom {\alpha 2}}\right.\kern-0pt} \!\lower0.7ex\hbox{$2$}}}}^{2} }}{2n} \pm z_{{{\raise0.7ex\hbox{$\alpha $} \!\mathord{\left/ {\vphantom {\alpha 2}}\right.\kern-0pt} \!\lower0.7ex\hbox{$2$}}}} \sqrt {\left[ {\hat{p}\left( {1 - \hat{p}} \right) + \frac{{z_{{{\raise0.7ex\hbox{$\alpha $} \!\mathord{\left/ {\vphantom {\alpha 2}}\right.\kern-0pt} \!\lower0.7ex\hbox{$2$}}}}^{2} }}{4n}} \right]/n} } \right)/\left( {1 + \frac{{z_{{{\raise0.7ex\hbox{$\alpha $} \!\mathord{\left/ {\vphantom {\alpha 2}}\right.\kern-0pt} \!\lower0.7ex\hbox{$2$}}}}^{2} }}{n}} \right)$$

where $$\hat{p}$$ = *X*/*n* is the sample proportion, *X* is a binomial variate for sample size *n*, and $$z_{{{\raise0.7ex\hbox{$\alpha $} \!\mathord{\left/ {\vphantom {\alpha 2}}\right.\kern-0pt} \!\lower0.7ex\hbox{$2$}}}}$$ is the 1 − $$\frac{\alpha }{2}$$ quantile of the standard normal distribution^29^. The observation of death was Bernoulli-sampled, and the likelihood function to estimate the case fatality risk *p* therefore used a binomial process (2):

2 $$L\;\left( {p_{t,a} ;\;X,\;\theta } \right) = \mathop \prod \limits_{i = 1}^{n} p_{t,a}^{{X_{i} }} \left( {1 - p_{t,a} } \right)^{{1 - X_{i} }}$$

where *p*_*t,a*_ is the case fatality risk of age group *a* at calendar time *t*, and *X*_*i*_ is the outcome for person *i* in age group *a* at calendar time *t* (*X*_*i*_ = 1 if died and 0 otherwise). For the interrupted time-series analysis, the case fatality risk at calendar time *t* was modelled as:

3 $$p_{t} = \beta_{0} + \beta_{1} T + \beta_{2} Y_{t(0)} + \beta_{3} Y_{t(1)} + \beta_{4} \left( {T - T_{0} } \right)Y_{t(0)} + \beta_{5} \left( {T - T_{1} } \right)Y_{t(1)}$$

where *T* is the time elapsed from the start of observation (i.e., equal to *t* − *t*_0_ where *t*_0_ is the clock zero of observation), *Y*_t(0)_ is the dichotomous variable representing the intervention as the state of emergency (i.e., 0 represents pre-emergency state and 1 is post-emergency state), and *Y*_t(1)_ is the dichotomous variable representing the intervention as the early diagnosis and treatment (i.e., 0 represents pre-intervention and 1 is post-intervention). *T*_0_ is the rescaled time at which the first intervention (i.e. the state of emergency) started, and *T*_1_ is the rescaled time at which intervention as early diagnosis and treatment started. $\beta_{0}$ is the parameter for the baseline level of the case fatality risk at rescaled time zero, $\beta_{1}$ measures the time-dependent increase in the case fatality risk, $\beta_{2}$ and $\beta_{3}$ correspond to change in the level of case fatality risk following first and second interventions, respectively. Similarly, $\beta_{4}$ and $\beta_{5}$ are the change in trend of case fatality risk following first and second interventions, respectively^30–32^. For the sensitivity analysis, we also considered models without particular parameters including $\beta_{4}$ and $\beta_{5} ,$ and compared the Akaike Information Criterion (AIC) to identify the best fit model. Parameters other than baseline time-trend, i.e., $\beta_{0} , \;\beta_{2} , \; \beta_{3} , \;\beta_{4} , \;{\text{and}}\;\beta_{5}$ were dealt with as age-dependent, and it was natural to assume that the trend parameter $\beta_{1}$ was shared by all age groups. Even for $\beta_{1}$, we compared models with $\beta_{1}$ being age-dependent and age-independent, using AIC. Maximum likelihood estimation was performed by minimizing the negative logarithm of (2), and the 95% confidence intervals (CIs) of the estimates were computed using the profile likelihood. The relative causal effects (i.e., the relative risk of death during the post-intervention period) of all groups over 50 years of age were estimated, using the case fatality risk on 7 May 2020 as the baseline. To identify regional differences, subgroup analysis was also conducted. To do so, we divided prefectures into two groups, i.e., prefectures with large population size and relatively high risk of infection, and reminder of prefectures. Prefectures with large population size was defined as seven prefectures in which the state of emergency was declared on 7 April 2020, including Tokyo, Kanagawa, Saitama, Chiba, Osaka, Hyogo, and Fukuoka.

### Ethical considerations

This study used only publicly announced data, and we did not handle any personally identifiable information. This study was approved by the Medical Ethics Board of the Graduate School of Medicine at Kyoto University (R2676). All methods were performed in accordance with the relevant guidelines and regulations.

### Data sharing statement

The original data were openly accessible from https://covid19.mhlw.go.jp/public/opendata/newly_confirmed_cases_daily.csv^33^ and are available as the Supplementary data.

## Results

**Figure 2:**
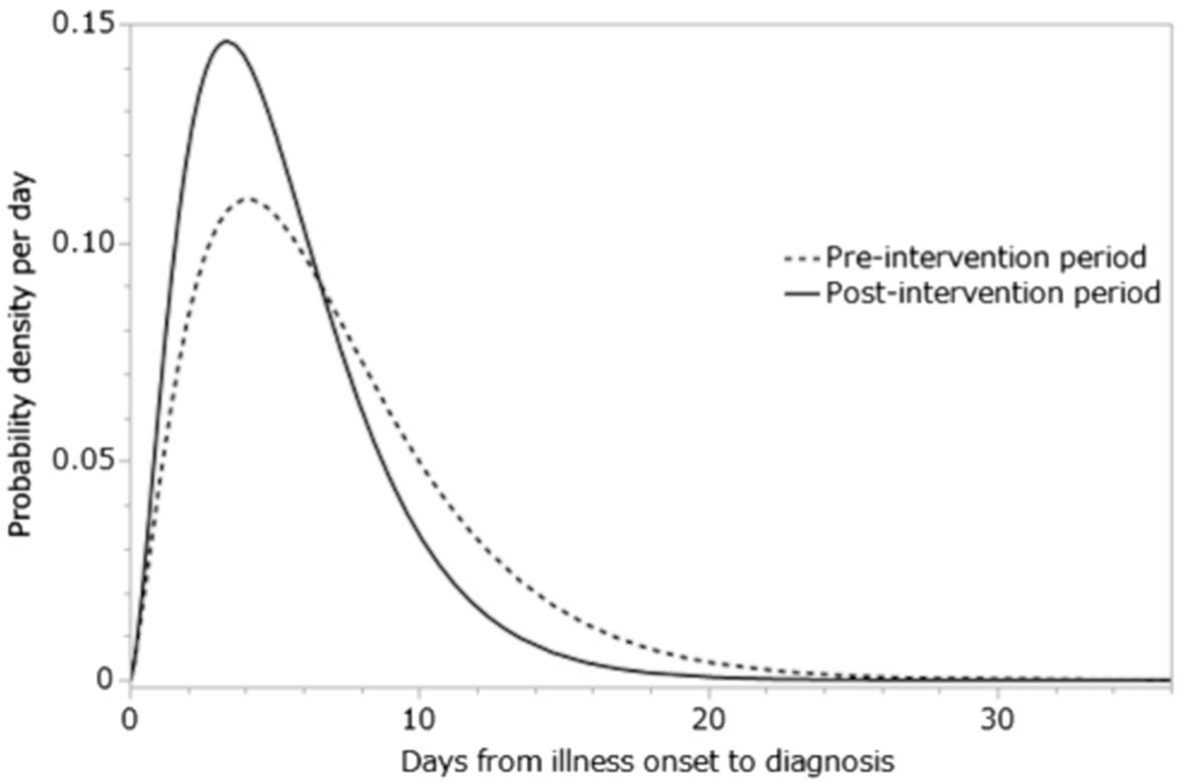
The probability density function of time delay from illness onset to diagnosis from 1 April to 15 June 2020 in Japan. The horizontal axis represents the days from illness onset to diagnosis. The vertical axis represents the probability density per day. The dotted line represents the pre-intervention period (i.e., before 8 May 2020), and the solid line the post-intervention period (i.e., on and after 8 May 2020).

From 14 March to 15 June 2020, a total of 7765 cases were confirmed, and of these, 11.7% died. The incidence of cases as a function of reporting date peaked in mid-April (Fig. 1), while deaths peaked in early May, reflecting the delay from illness onset to death. First, we examined the time delay distributions from illness onset to diagnosis, especially in relation to the delay before and after the date on which early diagnosis and treatment started. Figure 2 compares the fitted time delay from illness onset to diagnosis before and after 8 May 2020, divided into two groups by the date of illness onset. The average time delay from illness onset to diagnosis was 7.0 days and variance was 20.0 days^2^ among those who became ill before 8 May. The mean was shortened to 5.4 days and variance to 11.0 days^2^ on and after 8 May. Comparing the time delay between people who survived and died, the mean was 7.0 days and 6.3 days before 8 May, and dropped to 5.4 days and 5.3 days after 8 May.

**Figure 3:**
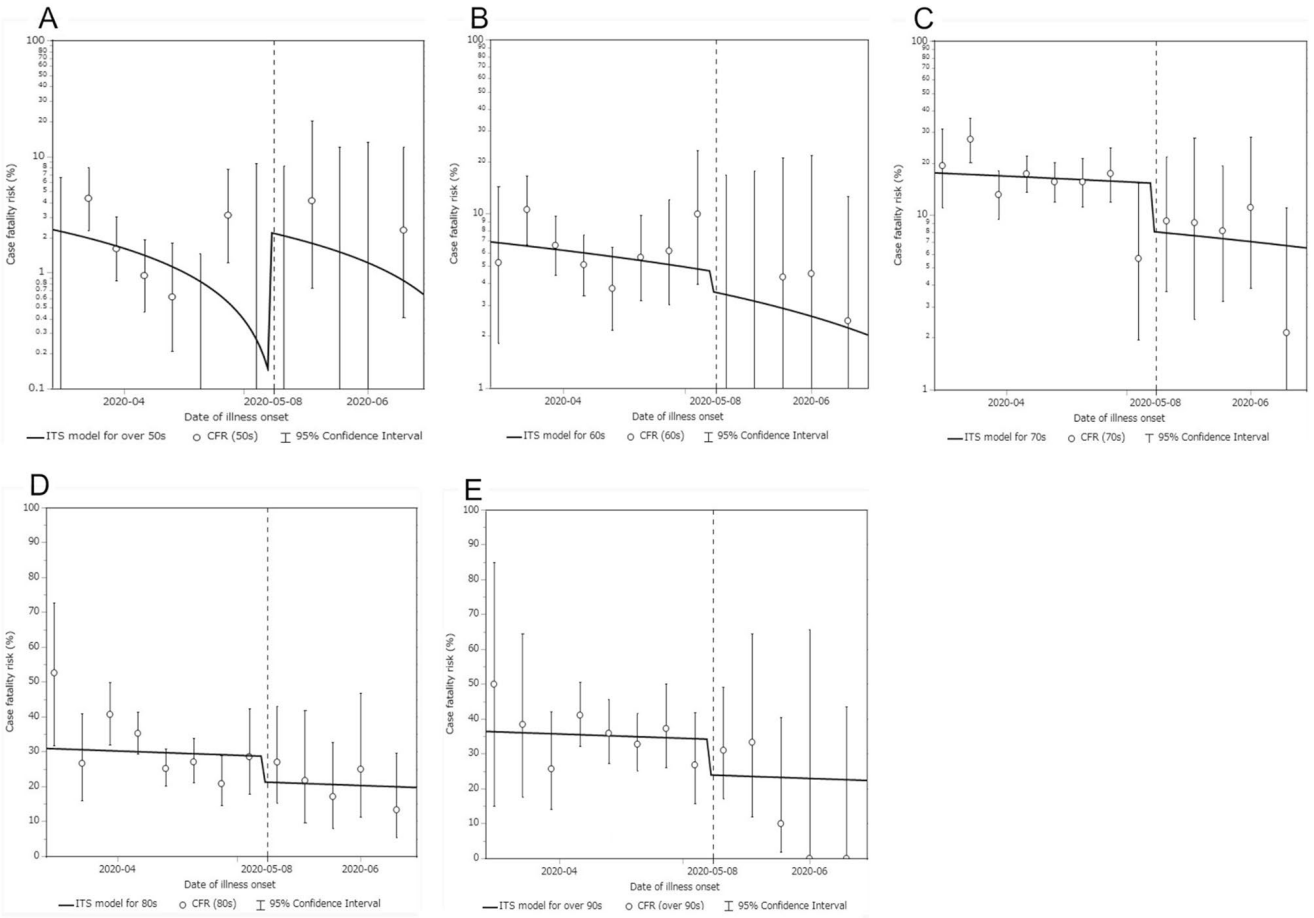
Case fatality risk of COVID-19 by age group from 1 April to 15 June 2020 in Japan. The vertical axis represents the case fatality risk. The horizontal axis represents the date of illness onset. The dotted line is the date on which the restriction on outpatient attendances was cancelled (i.e., people no longer had to wait 4 days from the onset of fever). The circular dots represent the empirically observed case fatality risk for every seven days, with error bars representing the 95% confidence intervals computed using Wilson’s score method. The upper left panel (**A**) shows the case fatality risk among cases aged 50–59 years, the upper middle panel (**B**) those aged 60–69 years, the upper right panel (**C**) those aged 70–79 years, the lower left panel (**D**) those aged 80–89 years, and the lower right panel (**E**) those aged 90 years and older.

Figure 3 shows the time dependent changes in the risk of death (case fatality risk) as a function of the date of illness onset. The fatality rate in every 10-year age group appeared to have decreased overall as a function of time, sharing a negative trend. Among those aged 60 years and older, there was an abrupt decline after 8 May 2020. The case fatality risk among those aged 50–59 years was generally lower than in older age groups, and the estimate slightly increased after 8 May. Table 1 summarizes the result of the model comparison. Comparing AIC values across examined models, the one that accounts only for early diagnosis and treatment as affecting the case fatality risk was yielded minimum AIC value. Namely, model 1, which accounts for an abrupt causal impact without a change in the trend after 8 May, was selected as the best fit model (AIC = 4638.77). Model 3, which accounts for an abrupt causal impact with a change in trend after 8 May, was the second-best model.

**Table 1:** Comparison of models that capture the time-dependent variations in the case fatality risk of COVID-19 in Japan.

Model parameters (see footnote for details)	Model 1* (With an abrupt causal effect and without trend change)	Model 2 (Without trend change and abrupt causal effect)	Model 3 (With an abrupt causal effect and trend change)	Model 4 (With trend change and without abrupt causal effect)
$\beta_{0}$	+	+	+	+
$\beta_{1}$	+	+	+	+
$\beta_{2}$	−	−	−	−
$\beta_{3}$	+	−	+	−
$\beta_{4}$	−	−	−	−
$\beta_{5}$	−	−	+	+
AIC	4638.77	4653.10	4640.59	4652.25

Plus shows what was accounted for, and minus that this was not included in that model. $\beta_{0}$ is the parameter for the baseline level; $\beta_{1}$, the increase in the outcome following the time-unit increase; $\beta_{2}$ and $\beta_{3}$, the change in the level at first and second interventions (7 April and 8 May 2020, respectively); and $\beta_{4}$ and $\beta_{5}$, the change in trend following first and second interventions (7 April and 8 May 2020, respectively).

Table 2 shows the parameter estimates of Models 1 and 3. The relative causal impact, i.e., the relative case fatality risk compared with the estimate on 8 May, was negative (i.e., the case fatality risk decreased during second intervention period) and estimated at − 24.3% for Model 1 and − 44.0% for Model 3 among those aged 60–69 years. Similarly, we estimated decreases of − 47.4% and − 50.6% among those aged 70–79 years, − 25.9% and − 27.6% among those aged 80–89 years, and − 30.1% and − 30.7% among those aged 90 and older.

**Table 2:** Parameter estimates of models to describe the time-dependent variations in the case fatality risk of COVID-19 in Japan.

Parameter descriptions	Model 1 (With an abrupt causal effect and without trend change); best model	Model 3 (With an abrupt causal effect and trend change); second best model
	Parameter estimates (95% confidence intervals)	
Intercept of 50 s, $\beta_{0\_50s}$ (%)	2.3 (1.4, 3.4)	2.4 (1.5, 3.4)
Intercept of 60 s, $\beta_{0\_60s}$ (%)	6.9 (5.6, 8.3)	6.9 (5.7, 8.3)
Intercept of 70 s, $\beta_{0\_70s}$ (%)	17.5 (15.6, 19.6)	17.5 (15.6, 19.6)
Intercept of 80 s, $\beta_{0\_80s}$ (%)	30.9 (28.1, 33.8)	30.9 (28.1, 33.9)
Intercept of over 90 s, $\beta_{0\_over90s}$ (%)	36.4 (32.2, 40.8)	36.4 (32.2, 40.8)
Baseline trend, $\beta_{1 }$ (% change per day)	− 0.04 (− 0.07, − 0.01)	− 0.04 (− 0.07, − 0.01)
Change in trend after intervention, $\beta_{5 }$ (% change per day)		0.03 (− 0.10, 0.17)
Causal impact on 50 s, $\beta_{3\_50s}$ (%)	2.1 (− 3.9, 3.0)	− 0.6 (− 12.6, 12.9)
Causal impact on 60 s, $\beta_{3\_60s}$ (%)	− 1.1 (− 3.9, 3.0)	− 3.9 (− 17.4, 10.3)
Causal impact on 70 s, $\beta_{3\_70s}$ (%)	− 7.2 (− 11.2, − 2.4)	− 9.6 (− 21.6, 2.7)
Causal impact on 80 s, $\beta_{3\_80s}$ (%)	− 7.4 (− 14.2, 0.2)	− 9.8 (− 22.9, 3.6)
Causal impact on over 90 s, $\beta_{3\_over 90s}$ (%)	− 10.3 (− 21.1, 2.7)	− 12.4 (− 0.1, 0.2)

$\beta_{0}$, the parameter for the baseline level; $\beta_{1}$, increase in the outcome following the time-unit increase; $\beta_{3}$, the change in the level at the intervention (8 May 2020); $\beta_{5}$, the change in trend following the intervention (8 May 2020).

In subgroup analysis, a total of 5367 and 2398 cases were confirmed, and of these, 12.2% and 10.4% died in seven large prefectures and remaining prefectures, respectively. Table 3 summarizes the result of model comparison. Among prefectures with large population sizes, Model 1 was again selected as the best fit model (AIC = 3306.83). Table 4 shows the result of the model comparison in remaining prefectures, and Model 4, which accounts for no abrupt causal impact without a change in trend after 8 May, appeared to be the best model (AIC = 1316.04).

**Table 3:** Comparison of models that capture the time-dependent variations in the case fatality risk of COVID-19 in seven urban prefectures in Japan.

Model parameters (see footnote for details)	Model 1* (With an abrupt causal effect and without trend change)	Model 2 (Without trend change and abrupt causal effect)	Model 3 (With an abrupt causal effect and trend change)	Model 4 (With trend change and without abrupt causal effect)
$\beta_{0}$	+	+	+	+
$\beta_{1}$	+	+	+	+
$\beta_{2}$	−	−	−	−
$\beta_{3}$	+	−	+	−
$\beta_{4}$	−	−	−	−
$\beta_{5}$	−	−	+	+
AIC	3306.82	3313.58	3308.75	3318.50

Plus shows what was accounted for, and minus that this was not included in that model. $\beta_{0}$ is the parameter for the baseline level; $\beta_{1}$, the increase in the outcome following the time-unit increase; $\beta_{2}$ and $\beta_{3}$, the change in the level at first and second interventions (7 April and 8 May 2020, respectively); and $\beta_{4}$ and $\beta_{5}$, the change in trend following the first and second interventions (7 April and 8 May 2020, respectively). Seven prefectures include Tokyo, Kanagawa, Saitama, Chiba, Osaka, Hyogo, and Fukuoka.

**Table 4:** Comparison of models that capture the time-dependent variations in the case fatality risk of COVID-19 in other than seven prefectures in Japan.

Model parameters (see footnote for details)	Model 1* (With an abrupt causal effect and without trend change)	Model 2 (Without trend change and abrupt causal effect)	Model 3 (With an abrupt causal effect and trend change)	Model 4 (With trend change and without abrupt causal effect)
$\beta_{0}$	+	+	+	+
$\beta_{1}$	+	+	+	+
$\beta_{2}$	−	−	−	−
$\beta_{3}$	+	−	+	−
$\beta_{4}$	−	−	−	−
$\beta_{5}$	−	−	+	+
AIC	1348.26	1316.04	1350.15	1316.56

Plus shows what was accounted for, and minus that this was not included in that model. $\beta_{0}$ is the parameter for the baseline level; $\beta_{1}$, the increase in the outcome following the time-unit increase; $\beta_{2}$ and $\beta_{3}$, the change in the level at first and second interventions (7 April and 8 May 2020, respectively); and $\beta_{4}$ and $\beta_{5}$, the change in trend following first and second intervention (7 April and 8 May 2020, respectively). Seven prefectures include Tokyo, Kanagawa, Saitama, Chiba, Osaka, Hyogo, and Fukuoka.

## Discussion

In Japan, the state of emergency was declared on 7 April 2020, and people were requested to stay at home. A restriction on outpatient attendances was imposed at the beginning of the first wave of the COVID-19 pandemic, and patients were asked to stay at home for at least four days after the onset of any fever. This restriction was cancelled on 8 May, and an antiviral, remdesivir (a nucleotide prodrug of an adenosine analog), was urgently approved and disseminated across Japan from 7 May. This study investigated the case fatality risk of COVID-19 before and after 7 April and 8 May 2020, exploring how the risk of death varied following promotion of early diagnosis and treatment. An interrupted time-series model was used to capture the time-dependent patterns of case fatality risk by age group during the first wave in Japan. Estimating the age-specific case fatality risk over the course of time showed a negative trend in this rate. We also found an abrupt decline in case fatality risk after 8 May. The causal impact, i.e., the abrupt decline in case fatality risk, was estimated as greater among older people.

To the best of our knowledge, this study is the first to have demonstrated in the public health observational setting that early diagnosis and treatment can abruptly and greatly improve the prognosis of COVID-19. The case fatality risk was halved immediately after the policy change among people aged from 70 to 79 years, potentially implying that about half of the people in these groups who would have died did not do so as a direct result of early diagnosis and treatment. The absolute decrease in the case fatality risk was greatest among people aged 90 years and older, i.e., the group most vulnerable to risk of death. We cannot separate the effects of treatment and early diagnosis, because both events took place during the time period of interest. However, the combination of early diagnosis and treatment gave a substantial improvement in outcomes. We have also shown that early diagnosis and treatment contributed to reducing case fatality risk, and this was not the case with suppressing infection with stay-at-home policy. However, it is true that the state of emergency has led to substantially reduce the number of infections and prevented healthcare facilities to be overwhelmed due to intense caseload demand. Exploring the similar subject in other settings would there be required to judge whether early diagnosis and treatment effectively reduce the risk of death in the absence of the state of emergency.

We also identified regional differences that influenced the case fatality risk. It is possible that the combination of early diagnosis and treatment contributed to substantial improvement of outcomes only in urban prefectures where transmission was intense. These prefectures were responsible for about a half of inpatients during the first wave^34,35^ and the 20% of designated beds were occupied from March to July 2020^36^. As there was a remaining capacity to admit patients, it is understandable that ensuring early diagnosis and treatment would have an effect in the urban setting. As well as reduced risk of death, we also identified a clear shortening in time delay from illness onset to diagnosis after 8 May. As an indicator of improved prognosis, the reduced mean number of days from illness onset to diagnosis indicates how fast infected individuals were provided with specific treatment and supportive care at healthcare facilities after 8 May, demonstrating that early diagnosis was indeed achieved. Regardless of prognosis, the delay from illness onset to diagnosis was shortened after 8 May. However, we found greater reduction among individuals who survived, implying that perhaps some of those who died before 8 May might not have done so if the diagnosis had been made earlier. As far as we are aware, there was no significant change in the number of PCR tests performed before and after 8 May^33^. Before the approval of antiviral, there was no specific treatment. The number of cases who were in need of medical attendance was decreasing during post-intervention period, and moreover, the antigen testing was first approved on 13 May 2020^37^. These imply that it is unlikely that limited capacity of diagnostic testing at the beginning of first wave led to a delay in diagnosis.

We have not shown the results from extensive sensitivity analyses, but the overall conclusions were not altered by those analyses. The alternative interrupted time-series models included alternative models (i) in which the time period of analysis was altered, i.e., instead of 1 April, we examined 18 and 25 March as the starting date, and instead of 15 June, we investigated 10, 20, 29, 30 June and 6 July as the last date, (ii) in which the date of intervention was altered, i.e., rather than 7 April and 8 May, we also examined 16 April and 12 May as the date of change, because the state of emergency was declared for all prefectures on 16 April 2020, and the issue of delayed diagnosis was discussed by the Minister of Health, Labour and Welfare and featured by mass media on that day, and (iii) in which the time trend in case fatality risk was not shared across different age groups. Even with those alterations, the abrupt decline in case fatality risk was identified among older people.

Four technical limitations must be discussed here. First, we did not directly measure how well the public was aware of the message about restrictions on outpatient attendances and then the abrupt policy change. However, a voluntary expert group on COVID-19 widely disseminated the message on this through social networks^38^, and “staying at home for 4 days after the onset of fever” was widely recognized, compared with other associated guidance. Second, we attributed the decline in case fatality risk to early diagnosis and treatment, but the treatment effect was not measured by comparing those who did and did not receive the treatment. We know that people with severe COVID-19 who received remdesivir had better outcomes^39,40^, but it was impossible to quantitatively attribute the causal impact to treatment alone. Third, it is possible that ascertainment bias could have varied over the course of the first wave, affecting statistical estimates of case fatality risk. However, there is no scientific evidence for this, and the testing volume did not greatly vary over time. Fourth, while we attributed the case fatality risk to early diagnosis and treatment, our study did not exclude the possibility of other explanatory factors, e.g., the change of characteristics of the patients during this period (increased recognition of the severity of disease), gradually increased number of medical facilities, and improved understanding of COVID-19 among physicians. In fact, the number of beds for COVID-19 increased from about 16,000 to 20,000 from May to July 2020^34,35^ and the guideline for the treatment of COVID-19 was updated three times from March to July 2020^41^.

Despite these limitations, this study suggests that early diagnosis and treatment substantially reduced the case fatality risk of COVID-19 among older people. The policy of encouraging early diagnosis and treatment is therefore vital to avoid as many deaths as possible from COVID-19.

## Supplementary Information

Supplementary Information.

## Data Availability

The original data were openly accessible from https://covid19.mhlw.go.jp/public/opendata/newly_confirmed_cases_daily.csv^28^ and are available as the Supplementary data.
